# Consumer Awareness and Acceptance of Palm Weevil Larvae (*Rhynchophorus phoenicis*) for Use in Processed Meat Products

**DOI:** 10.1155/ijfo/9930346

**Published:** 2026-06-27

**Authors:** Mercy Amo Aggrey, Worlah Yawo Akwetey

**Affiliations:** ^1^ Department of Animal Science, Faculty of Agriculture, College of Agriculture and Natural Resources, Kwame Nkrumah University of Science and Technology (KNUST), Kumasi, Ghana, knust.edu.gh

**Keywords:** akokono, alternative protein source, consumer perception, edible insects, processed meat products

## Abstract

Palm weevil larvae (*Rhynchophorus phoenicis*), locally known as akokono, are traditionally consumed in Ghana but remain inadequately integrated into commercialized food systems. This study assessed consumer awareness of akokono as a food source, willingness of nonconsumers to try processed akokono products, and acceptance of akokono as an ingredient in processed meat products in Ayeduase, Kumasi, Ghana. A cross‐sectional survey design involving 150 respondents selected through a mixed convenience–intercept and snowball sampling approach was employed. Data were collected using a structured questionnaire and analyzed using descriptive statistics and chi‐square tests in IBM SPSS Statistics Version 27. The results showed that 41.3% of respondents currently consumed akokono, with taste identified as the major reason for consumption (80.6%). Although regular consumption was moderate, awareness of akokono as a traditional food source was generally high among respondents. Among nonconsumers, 58.8% expressed willingness to consume akokono when incorporated into processed products, while sausage was the most preferred product form (59.7%). Acceptance of processed akokono products was generally favorable, particularly when incorporated into familiar food forms. Chi‐square analysis revealed no significant association between akokono consumption and sociodemographic variables, including gender, age, and educational level (*p* > 0.05). The findings suggest that incorporating akokono into familiar processed food products may improve consumer acceptance and support the promotion of sustainable alternative protein sources.

## 1. Introduction

The increasing global demand for sustainable and alternative protein sources has intensified interest in edible insects as potential contributors to future food systems.

Rising population growth, increased pressure on conventional livestock production, and environmental concerns associated with meat production have heightened the need to diversify protein sources [1, 2]. Compared with conventional livestock, edible insects require fewer environmental resources and have lower greenhouse gas emissions, making them promising candidates for sustainable food production systems [3].

Edible insects have long been consumed in many parts of sub‐Saharan Africa and contribute significantly to household nutrition, food security, and livelihoods. In Ghana, palm weevil larvae (*Rhynchophorus phoenicis*), locally known as akokono, are among the most commonly consumed edible insects. Traditionally, akokono is consumed roasted, fried, or boiled and is valued for its distinctive flavor and cultural importance [4, 5]. Nutritionally, palm weevil larvae are rich in high‐quality protein, essential fatty acids, and micronutrients, suggesting strong potential for use in food product development and dietary diversification [6, 7].

Despite their nutritional and cultural significance, the consumption of akokono in Ghana remains largely informal and concentrated in traditional forms. The incorporation of palm weevil larvae into processed food systems, particularly processed meat products, remains limited and underexplored. Existing studies on edible insects have primarily focused on nutritional composition, environmental sustainability, and general consumer perceptions of entomophagy, with relatively limited attention given to consumer acceptance of palm weevil larvae incorporated into familiar processed food products within Ghanaian settings [1, 8].

Consumer acceptance remains one of the major barriers to the commercialization of insect‐based foods. Food neophobia, perceived safety concerns, cultural attitudes, and aversion to visible insect forms have been identified as significant constraints to adoption, especially among urban consumers [9–11]. However, previous studies have shown that acceptance tends to improve when insects are incorporated into familiar and processed food matrices that reduce visibility and align with existing dietary habits [12, 13]. Processed meat products such as sausages may therefore provide a practical vehicle for improving consumer acceptance of edible insect ingredients.

Although studies on edible insects are increasing globally, empirical data on consumer awareness, willingness, and acceptance of akokono incorporated into processed meat products remain scarce in Ghana, particularly within urban and periurban populations. Understanding consumer perceptions toward such products is important for guiding product development, commercialization strategies, and policy discussions surrounding sustainable alternative proteins.

Therefore, this study assessed consumer awareness of akokono as a food source, willingness of nonconsumers to try processed akokono products, and acceptance of akokono as an ingredient in processed meat products in Ayeduase, Kumasi, Ghana. The study further examined consumption patterns and the influence of sociodemographic factors on akokono consumption and acceptance.

## 2. Materials and Methods

### 2.1. Study Area

The study was carried out in Ayeduase, a suburb of Kumasi in the Ashanti Region of Ghana. Ayeduase, which shares a boundary with Kwame Nkrumah University of Science and Technology (KNUST), was chosen due to its proximity to KNUST. It features a heterogeneous population of students, staff, traders, and native Asantes, as well as relevance as an early market opportunity for novel food products.

### 2.2. Study Design and Population

This study employed a cross‐sectional survey design to assess the consumer awareness and usage of akokono. The target population was the youth and adult consumers residing in Ayeduase, Kumasi.

### 2.3. Sample Size and Sampling Technique

A total of 150 respondents participated in the study. The sample size was determined using Cochran′s sample size formula for exploratory survey studies, using a 50% assumed prevalence level to maximize variability, a 95% confidence level, and a 5% margin of error. The calculated minimum sample size was approximately 384 respondents; however, due to time, logistics, and resource constraints associated with this exploratory study, a sample of 150 respondents was considered adequate for generating preliminary insights into consumer awareness and acceptance of akokono‐based processed meat products. This approach was considered appropriate due to the limited availability of prior data on consumer acceptance of akokono in processed meat products within the study area.

A mixed nonprobability sampling approach comprising convenience–intercept and snowball sampling techniques was employed. Convenience–intercept sampling was used to recruit respondents from public locations such as markets, shops, food vending areas, and transport stations within Ayeduase, where diverse groups of consumers could be accessed. Snowball sampling was subsequently used to recruit additional participants through referrals from initial respondents, particularly individuals familiar with akokono consumption.

The combined sampling approach was considered appropriate for this exploratory study because processed akokono products remain relatively novel within the study population, and limited prior data exist on consumer acceptance of such products in Ghana. Nevertheless, the use of nonprobability sampling may limit the representativeness and wider generalizability of the findings. Consequently, the results should be interpreted as reflecting consumer perceptions within the study area and similar urban and periurban settings.

### 2.4. Data Collection Instrument

Data were collected using a structured questionnaire comprising both closed‐ and open‐ended questions. The questionnaire was designed based on the objectives of the study and relevant literature on consumer perception and acceptance of edible insects. The instrument collected information on sociodemographic characteristics, awareness and current consumption of akokono, sources and forms of acquisition, reasons for consumption, willingness to consume processed akokono products, and preferences for processed product forms. Perception‐related items assessed respondents′ acceptance of akokono incorporated into processed meat products and factors influencing willingness to consume such products.

Prior to the main survey, the questionnaire was pretested among 15 individuals with demographic characteristics similar to those of the target population, but they were not included in the final study sample. The pretest was conducted to evaluate the clarity, relevance, sequencing, and comprehensibility of the questionnaire items. Feedback obtained from the pretest was used to refine question wording, improve clarity, and ensure consistency before the commencement of the main data collection exercise.

Data collection was conducted through face‐to‐face administration of the questionnaire.

This study adhered to standard ethical guidelines for research involving human participants. Participation was voluntary, and informed consent was obtained from all respondents prior to data collection. No personal identifying information was collected, and all responses were treated with strict confidentiality. The study posed minimal risk to participants and complied with institutional ethical standards for research. Formal ethical clearance was not required for this study because it involved anonymous questionnaire‐based data collection with no invasive procedures or identifiable personal information.

### 2.5. Statistical Analysis

Completed questionnaires were checked for completeness, coded, and analyzed using IBM SPSS Statistics Version 27. Descriptive statistics (frequency and percentages) were used to summarize demographic variables, awareness levels, consumption patterns, willingness to consume processed akokono products, and consumer preferences.

Inferential statistical analysis was conducted using the chi‐square test of independence to examine associations between selected sociodemographic variables (gender, age group, and educational level) and akokono consumption. The chi‐square test was considered appropriate because the variables analyzed were categorical in nature. The null hypothesis stated that there was no significant association between sociodemographic variables and akokono consumption, whereas the alternative hypothesis stated that a significant association existed.

Statistical significance was determined at a 95% confidence level using a significance threshold of *p* < 0.05. Assumptions underlying the chi‐square test, including independence of observations and adequacy of expected cell frequencies, were considered during the analysis. Given the exploratory nature of the study and the categorical structure of the variables collected, the analysis focused primarily on descriptive statistics and chi‐square tests of association. Although multivariate analytical approaches such as logistic regression could provide deeper insights into predictors of consumer acceptance, such analyses were beyond the scope of the present exploratory study. Future studies employing larger and more representative samples may incorporate advanced multivariate models to further examine relationships among awareness, perceptions, willingness, and acceptance of insect‐based food products.

## 3. Results and Discussion

### 3.1. Demographic Characteristics

Table 1 shows respondents comprised 52% males and 48% females, with most aged between 31 and 56 years. Educational levels varied, with 32.7% having tertiary education and only 9.3% of respondents being without education. Most respondents had some level of formal education, ranging from JHS to tertiary level.

**Table 1:** Demographic profile of respondents (*N* = 150).

Variable	Category	Frequency (*n*)	Percentage (%)	Valid % (adjusted)
**Gender**	Male	78	52.0	52.0
	Female	72	48.0	48.0
	**Total**	**150**	**100.0**	**100.0**
**Age group (years)**	18–30	36	24.0	24.0
	31–43	47	31.3	31.3
	44–56	45	30.0	30.0
	57–69	22	14.7	14.7
	**Total**	**150**	**100.0**	**100.0**
**Education level**	Not educated	14	9.3	9.3
	JHS	42	28.0	28.0
	SHS	45	30.0	30.0
	Tertiary	49	32.7	32.7
	**Total**	**150**	**100.0**	**100.0**

Abbreviations: JHS, junior high school; SHS, senior high school.

### 3.2. Awareness and Consumption Patterns

The findings showed that awareness of palm weevil larvae (akokono) as a traditional food source was relatively high among respondents, reflecting its long‐standing cultural relevance within Ghanaian diets. This suggests that edible insects continue to contribute to indigenous food systems and household nutrition in many African communities, consistent with findings reported by Kelemu et al. [3] and van Huis et al. [2]. Cultural familiarity may therefore play an important role in shaping awareness and reducing initial resistance toward insect‐based foods.

Despite the relatively high awareness levels, regular consumption of akokono was comparatively moderate, with only 41.3% of respondents indicating current consumption (Table 2). This disparity between awareness and actual consumption suggests that familiarity alone may not necessarily translate into regular dietary adoption. Urbanization, modernization, and changing dietary lifestyles may partly explain this trend, as consumers increasingly shift toward processed and convenience foods while gradually moving away from certain traditional food practices [14]. The findings further imply that although akokono remains culturally recognized, contemporary consumer preferences may increasingly favor products that are convenient, standardized, and compatible with modern eating habits (Table 3). This may explain why respondents demonstrated greater willingness to consume akokono when incorporated into processed products rather than in its traditional whole form. Similar observations have been reported in edible insect studies where processing and product familiarity contributed positively to consumer acceptance by reducing psychological barriers associated with insect consumption [9, 12].

**Table 2:** Consumption status (*N* = 150).

Variable	Category	Frequency (*n*)	Percentage (%)
**Consume akokono currently**	Yes	62	41.3
	No	88	58.7
	**Total**	**150**	**100.0**

Abbreviations: %, percentage of respondents; n, number of respondents.

**Table 3:** Behavioral characteristics of current akokono consumers (*n* = 62).

Variable	Category	Frequency (*n*)	Percentage (%)
**Where akokono is obtained (for current consumers)**	Free from farm	41	66.1
	Open market	12	19.4
	Akokono house	7	11.3
	Other sources	2	3.2
	**Total**	**62**	**100.0**
**Form of purchase (for current consumers)**	Raw	33	53.2
	Khebab	23	37.1
	Shito	4	6.5
	Spread	2	3.2
	**Total**	**62**	**100.0**
**Reason for consumption (for current consumers)**	Taste	50	80.65
	Alternative protein source	5	8.06
	Health benefits	7	11.29
	**Total**	**62**	**100.0**
**Typical mealtimes of consumption**	Breakfast	2	3.2
	Lunch	37	59.7
	Dinner	16	25.8
	Others	7	11.3
	**Total**	**62**	**100.0**
**Preferred akokono product**	Sausage only	37	59.7
	Baked loaf only	4	6.5
	Both sausage and loaf	21	33.9
	**Total**	**62**	**100.0**

*Note:* Percentages were calculated based on valid responses from current akokono consumers only (*n* = 62).

Abbreviations: %, percentage of respondents; n, number of respondents.

### 3.3. Acceptance of Akokono in Processed Meat Products

The findings showed that many respondents, particularly nonconsumers, expressed willingness to consume akokono when incorporated into processed meat products. This suggests that product form plays an important role in influencing consumer acceptance of edible insects. Processed food formats such as sausages may reduce negative perceptions associated with the visible appearance of insects and thereby improve willingness to try insect‐based foods (Table 4).

**Table 4:** Willingness to consume akokono when processed (nonconsumers only; *n* = 88).

Variable	Category	Frequency (*n*)	Percentage (%)
**Willing to consume when processed (if nonconsumer)**	Yes	50	58.8
	No	36	40.9
	No responses	2	2.3
Total		**88**	**100**

Abbreviations: %, percentage of respondents; n, number of respondents.

The willingness of many known consumers to try akokono in processed products may be attributed to the concealment of visible insect characteristics within familiar food metrices. Product concealment reduces feelings of disgust and uncertainty often associated with whole insect consumption while increasing perceptions of familiarity and convenience [12, 15]. This observation aligns with previous studies reporting improved acceptance of edible insects when incorporated into food products such as sausages, burgers, and snacks.

Food neophobia, defined as reluctance to consume unfamiliar foods, has consistently been identified as a major barrier to edible insect acceptance, especially among urban consumers and younger populations [9, 11]. In the present study, acceptance appeared to improve when akokono was incorporated into familiar food matrices, suggesting that processing may help minimize psychological barriers such as disgust and fear associated with whole insect consumption. Sensory perception may also have contributed to the observed acceptance patterns. Taste was identified as the primary reason for current consumption among existing consumers, indicating that positive eating experiences may encourage continued acceptance of akokono products. Previous studies have similarly reported that favorable sensory characteristics, including flavor and texture, significantly influence consumer willingness to adopt edible insect‐based foods [12].

Cultural familiarity further appears to play an important role in shaping acceptance. Because akokono is already traditionally consumed in parts of Ghana, respondents may perceive processed akokono products as less culturally unfamiliar compared with completely novel insect foods. Comparable studies in Kenya and Uganda have also shown that consumer acceptance improves when edible insects are incorporated into familiar processed foods and culturally accepted dishes [13, 16].

These findings suggest that successful commercialization of akokono‐based products may depend not only on nutritional value but also on effective product formulation strategies that address sensory expectations, reduce food neophobia, and align with existing cultural food practices.

### 3.4. Awareness of Nutrition and Safety

Although most respondents were aware of akokono as a traditional food source, relatively few demonstrated awareness of its nutritional importance and food safety considerations. This knowledge gap may influence consumer perceptions and limit wider acceptance of akokono‐based processed products.

Palm weevil larvae are recognized as nutrient‐dense foods containing high‐quality protein, essential fatty acids, and important micronutrients such as iron and zinc [7]. Studies have also shown that edible insect oils contain nutritionally valuable fatty acids that may contribute positively to human nutrition [6]. Despite these nutritional benefits, consumer acceptance of insect‐based foods is strongly influenced by perceptions regarding hygiene, processing safety, and product quality.

Food safety concerns remain an important challenge in the commercialization of edible insect products. Consumers may be hesitant to adopt insect‐based foods due to concerns regarding contamination, microbial safety, allergenicity, and hygienic processing practices. According to van Huis et al. [2], edible insects can be considered safe for human consumption when harvested, processed, stored, and handled under appropriate hygienic conditions. Proper thermal processing, packaging, and storage are therefore essential to ensure product safety and maintain consumer confidence.

Processing also has important implications for product quality and market acceptance. Incorporating akokono into processed meat products may improve standardization, shelf stability, and convenience while reducing the visibility of insect components that often contribute to consumer rejection. However, successful commercialization will require adherence to food safety standards, quality assurance systems, and appropriate regulatory frameworks governing insect‐based foods.

In Ghana and many developing countries, regulatory systems specific to edible insects remain limited or underdeveloped. Strengthening public education, food safety guidelines, and regulatory oversight may therefore be necessary to support the safe production, commercialization, and consumer acceptance of akokono‐based processed products.

### 3.5. Influence of Sociodemographic Factors

Chi‐square analysis (Table 5) showed no statistically significant association between akokono consumption and respondents′ gender (*χ* ^2^ = 1.557, *p* = 0.212), age group (*χ* ^2^ = 9.762, *p* = 0.082), or educational level (*χ* ^2^ = 7.790, *p* = 0.051). These findings suggest that consumption and acceptance of akokono within the study population were not primarily determined by sociodemographic characteristics. Instead, consumer education, product quality, sensory appeal, and effective marketing strategies may be more important determinants of acceptance across diverse consumer groups.

**Table 5:** Association between sociodemographic characteristics and akokono consumption among respondents (*n* = 150).

Variable		Consumption of akokono		Total	X^2^	df.	*p*
		No	Yes				
Gender	Female	46	26	72	1.557	1	0.212
	Male	42	36	78			
	**Total**	**88**	**62**	**150**			
Age	18–30	26	9	35	9.762	5	0.082
	31–43	22	22	44			
	44–46	3	0	3			
	47–56	23	15	38			
	57–69	14	15	29			
	70 above	0	1	1			
	**Total**	**88**	**62**	**150**			
Level of education	JHS	27	14	41	7.790	3	0.051
	SHS	24	16	40			
	Not educated	3	10	13			
	Tertiary	34	22	56			
	**Total**	**88**	**62**	**150**			
Consumption of processed akokono	No	37			a	a	a
	Yes	50		37			
	**Total**	**87**		**50**			

*Note:* “a” denotes no statistics are computed because consumption of akokono is a constant. Statistical significance was determined at *p* < 0.05.

Abbreviations: *χ* ^2^, chi‐square statistic; df, degrees of freedom; p, probability value.

The absence of statistically significant relationships indicates that acceptance of akokono may be more strongly influenced by product‐related factors such as processing form, familiarity, accessibility, and perceived nutritional benefits rather than demographic differences. Similar observations have been reported in previous edible insect studies, where product presentation and reduction of visible insect characteristics significantly improved consumer acceptance irrespective of demographic background [9, 11].

Although educational attainment is often associated with openness toward novel foods, the present findings suggest that education alone may not necessarily determine willingness to consume insect‐based products. Comparable studies have also reported inconsistent associations between educational level and edible insect consumption behavior [1].

### 3.6. Sampling Considerations and Generalizability

The findings of this study should be interpreted in light of certain limitations. Therefore, the findings should not be interpreted as representative of all Ghanaian consumers but rather of consumers within Ayeduase and similar urban or periurban settings. The study employed convenience–intercept and snowball sampling techniques, which are nonprobability sampling methods commonly used in exploratory consumer perception research. Although these approaches facilitated access to respondents familiar with akokono consumption, they may have introduced selection bias and limited the representativeness of the sample.

Additionally, the study relied on self‐reported responses, which may be influenced by recall bias, social desirability bias, or respondents′ subjective perceptions. Consequently, participants′ reported awareness, consumption patterns, and willingness to consume processed akokono products may not fully reflect actual consumer behavior.

The geographic scope of the study was limited to Ayeduase in Kumasi, Ghana. Consumer perceptions and acceptance patterns may differ across regions, ethnic groups, and rural or urban populations within the country. Therefore, the findings should be interpreted cautiously and should not be generalized to the entire Ghanaian population.

Despite these limitations, the study provides important preliminary insights into consumer awareness, willingness, and acceptance of akokono in processed meat products within an urban and periurban Ghanaian context. Future studies employing probability‐based sampling techniques, larger sample sizes, broader geographic coverage, and advanced multivariate analyses are recommended to improve representativeness and strengthen generalizability.

### 3.7. Implications for Product Development

Overall, results indicate that akokono has a strong cultural legitimacy that could facilitate its incorporation into processed meat products. The willingness to consume akokono in processed form currently, coupled with the lack of demographic barriers, are good indicators for product development.

However, successful commercialization will hinge upon mitigating consumer fears around safety, quality assurance, and nutritional literacy. Through the communication of strategic nutrition around protein and micronutrient benefits of palm weevil larvae, this study also contributes to additional bases for enhancing consumer acceptance toward sustainable alternative protein products.

## 4. Conclusion and Recommendation

This study assessed consumer awareness, willingness, and acceptance of palm weevil larvae (akokono) as an ingredient in processed meat products in Ayeduase, Kumasi, Ghana. The study was undertaken in response to the limited information available on consumer perceptions of akokono incorporation into processed foods despite growing interest in edible insects as sustainable alternative protein sources. The findings indicated that the awareness of akokono as a traditional food source was relatively high, although current consumption levels were moderate. A considerable proportion of nonconsumers expressed willingness to consume akokono when incorporated into processed food products, particularly sausages. In addition, no statistically significant association was observed between akokono consumption and sociodemographic variables such as gender, age, and educational level.

The results also suggest that incorporating akokono into familiar processed food forms may improve consumer receptiveness toward edible insect‐based products within similar urban and periurban populations. However, acceptance appears to be influenced by factors such as product form, sensory perception, familiarity, and food safety considerations rather than sociodemographic characteristics alone. Although the study provides useful preliminary insights into consumer perceptions of processed akokono products, the findings should be interpreted within the limitations of the study design and sampling approach. Further studies involving larger and more representative populations, sensory evaluations, and market‐based assessments are recommended to better understand the commercialization potential of akokono‐based processed food products in Ghana.

Overall, the study demonstrates that incorporating akokono into familiar food products may provide a practical strategy for increasing consumer acceptance of edible insects. Product development efforts that emphasized sensory quality, food safety, and consumer education may enhance market acceptance and contribute to the promotion of sustainable alternative protein sources in Ghana.

## Funding

No funding was received for this manuscript.

## Conflicts of Interest

The authors declare no conflicts of interest.

## Data Availability

The data that support the findings of this study are available on request from the corresponding author. The data are not publicly available due to privacy or ethical restrictions.
